# Effect of hemodialysis on high-sensitivity cardiac troponin T levels in patients with hypervolemia

**DOI:** 10.3389/fneph.2025.1717448

**Published:** 2025-12-18

**Authors:** Mohammad Tinawi, Bahar Bastani

**Affiliations:** 1 Department of Medicine, Indiana University School of Medicine Northwest-Gary, Gary, IN, United States; 2 Department of Internal Medicine, Saint Louis University, St. Louis, MO, United States

**Keywords:** acute coronary syndrome, cardiac troponins, end-stage kidney disease, hemodialysis, hypervolemia

## Abstract

**Background:**

High-sensitivity cardiac troponin T (hs-cTnT) is widely used in the diagnosis of acute coronary syndrome (ACS) because it is a marker of myocardial damage. Most patients with end-stage kidney disease (ESKD) on renal replacement therapy have elevated plasma hs-cTnT levels at baseline. The impact of hemodialysis (HD) on hs-cTnT levels remains unclear. This study aimed to determine the effect of HD in patients with ESKD and hypervolemia on plasma hs-cTnT levels.

**Methods:**

We conducted a retrospective study of ESKD patients admitted to two community hospitals over a three-year period (from January 1, 2020, to December 31, 2022). All patients had hypervolemia on admission. Plasma hs-cTnT levels were measured at admission and repeated 5.5 ± 0.75 hours after HD. Over the study period, 20 patients with ESKD and hypervolemia fulfilled the inclusion criteria. Two patients were diagnosed with ACS.

**Results:**

Pre-HD and post-HD hs-cTnT were elevated in 85% of patients. The data did not follow normal distribution. The median and interquartile range (IRQ) for pre-HD hs-cTnT was 126 (154) ng/L, and for post-HD hs-cTnT was 155 (234) ng/L. Following a single HD session with a high-flux dialyzer, hs-cTnT levels increased in 80% of the cohort, with a mean rise of 25.6% (p = 0.0042). Mean volume removal was 2.4 L, range (1–5 L). Two patients were diagnosed with ACS. Mortality over the study period was 40%, with cardiovascular disease as the leading cause of death.

**Conclusion:**

In ESKD patients with hypervolemia, a single HD session using a high-flux dialyzer significantly increased hs-cTnT plasma level. Pre-dialysis hs-cTnT measurements should be used as a clinical baseline when evaluating for ACS, and post-dialysis elevations should be interpreted with caution. Serial measurements may improve diagnostic accuracy. Further prospective studies are needed to clarify the mechanisms and clinical implications of these findings.

## Introduction

Troponins are protein complexes found in skeletal muscle and myocardial cells. They play a pivotal role in muscular contraction. There are three different types of troponins: Troponin C (TnC), troponin I (TnI), and troponin T (TnT). Myocardial injury releases cardiac troponin I (cTnI) and cardiac troponin T (cTnT). High-sensitivity assays of cTnI and cTnT are routinely utilized to diagnose myocardial ischemia due to their high sensitivity and specificity (1). The half-lives of cTnT and cTnI are around 2 hours, but damaged myocardium continues to release cardiac troponin resulting in an apparent half-life of up to 24 hours (2). Animal studies revealed that cardiac troponins are cleared by receptor-mediated endocytosis in the liver and the kidney. Moreover, they are amenable to degradation by proteolysis in the bloodstream and in cardiac myocytes. The resulting smaller fragments can be filtered by the kidney (2).

Many asymptomatic patients with chronic kidney disease (CKD) have elevated plasma levels of cardiac troponins at baseline without clear evidence of myocardial ischemia based on currently available diagnostic studies (3). In asymptomatic patients with end-stage kidney disease (ESKD), cTnT levels are elevated more frequently than cTnI levels (4). Such increases are associated with adverse outcomes including all-cause mortality and sudden cardiac death (5, 6). An elevated cTnT plasma level is associated with poor short-term prognosis irrespective of creatinine clearance, making it a specific marker of ACS in all patients including those with CKD (7). HD can affect the levels of cardiac troponins. Studies on this issue have yielded conflicting data. In theory, levels of cardiac troponin can increase due to fluid removal via ultrafiltration resulting in hemoconcentration or decrease due to clearance by dialysis or binding to the dialyzer. The rate of ultrafiltration, and the type of dialyzer (low-flux vs high-flux) may have an effect. The molecular weight of troponin I and T is approximately 24 kDa, and 35 kDa, respectively. Low-flux dialysis membranes can clear molecules up to 5 kDa, while high-flux membranes can clear molecules up to 20 kDa. Medium cut-off (MCO) membranes are more porous and can clear larger molecules up to 45 kDa (8).

The authors have recently published a report on the impact of volume removal by HD on elevated N-terminal pro-brain-type natriuretic peptide (NT-proBNP) levels in patients with hypervolemia (9). The report concluded that volume removal via a single HD session had no impact on NT-proBNP level, and that repeating NT-proBNP levels post-HD had no clinical value. An elevated level of NT-proBNP predicted high cardiovascular mortality and recurrent hospitalizations in acute and chronic HD patients. Of the 41 patients included in that report, 20 patients had pre- and post-HD hs-cTnT plasma level measurements, and they are the subject of this analysis. The purpose of this retrospective analysis is to determine the effect of HD with high-flux dialyzer on hs-cTnT plasma levels in patients with ESKD and hypervolemia.

## Methods

**Figure 1:**
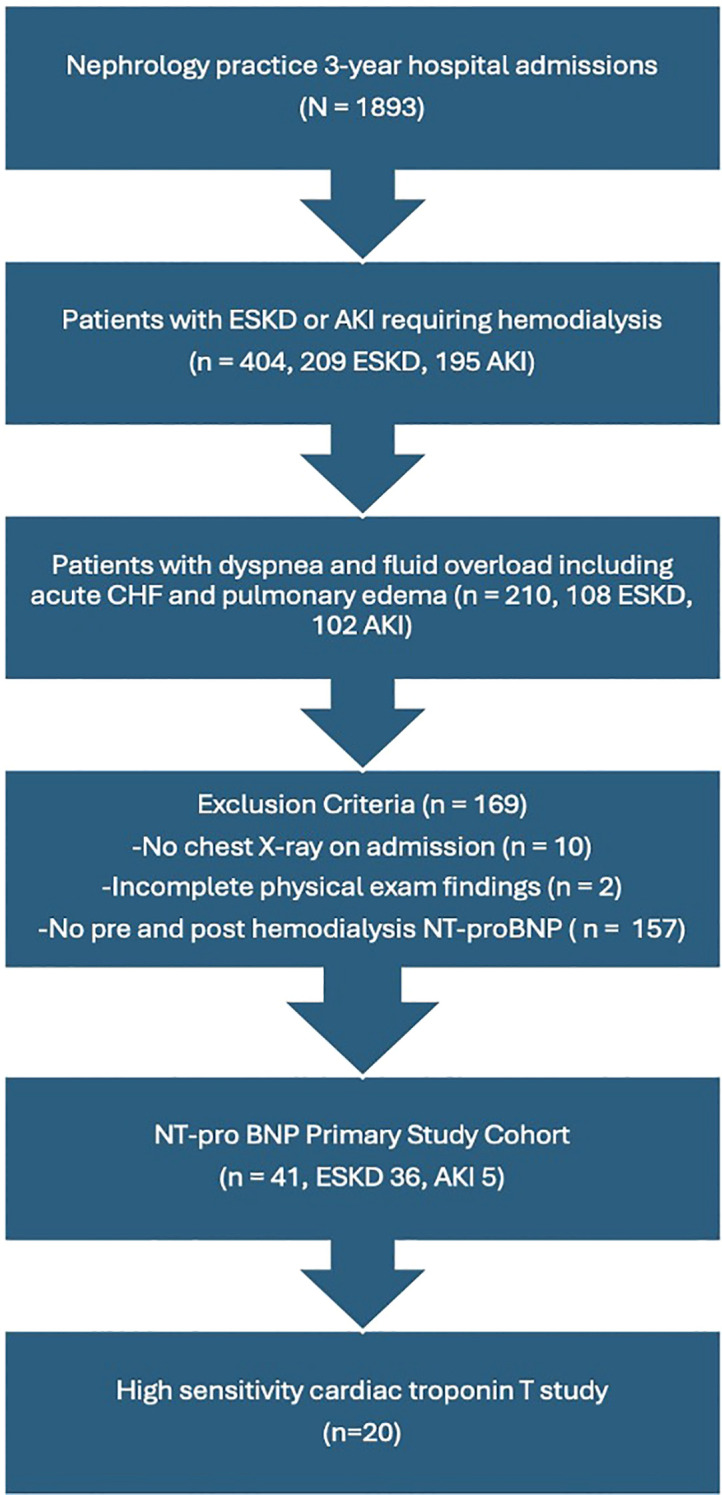
Flowchart illustrating the selection of the study population. Of 1,893 total admissions, 210 patients with ESKD or AKI presented with dyspnea and hypervolemia. After applying exclusion criteria, 41 patients were included in the NT-proBNP cohort, of whom 20 had both pre- and post-HD hs-cTnT measurements and comprised the final study population. CHF, congestive heart failure; NT-proBNP, N-terminal pro-brain-type natriuretic peptide.

An institutional review board (IRB) approval was obtained; and the study was conducted in compliance with all the applicable institutional ethical guidelines. This is an analysis of a retrospective cohort admitted by a single-specialty nephrology group to two community hospitals over a three-year interval (January 1, 2020-December 31, 2022). The goal of this retrospective analysis was to evaluate the effect of a single HD session via a high-flux dialyzer membrane on hs-cTnT plasma levels in patients with ESKD with hypervolemia. Patients were included if they were admitted with a diagnosis of acute kidney injury (AKI) or ESKD irrespective of dialysis vintage. All patients had dyspnea on admission and physical examination with radiographic evidence of hypervolemia including acute congestive heart failure (CHF) or overt pulmonary edema. All patients had a chest radiograph showing increased pulmonary vascular congestion or pulmonary edema. All patients had hypervolemia signs on physical examination such as crackles on lung examination and lower extremities pitting edema. Patients had plasma NT-proBNP levels drawn upon admission (less than 24 hours before their first inpatient HD session), and a repeat measurement 2–12 hours after the same HD treatment. Additionally, patients had hs-cTnT measured on admission and a repeat measurement post-HD session, 5.5 ± 0.75 hours from the initial measurement. Patients were not included if their physical examination findings were incomplete or if they did not have a chest radiograph on admission. Over the 3-year period 108 ESKD patients and 102 AKI patients were admitted with dyspnea and hypervolemia, of whom 36 patients with ESKD and 5 patients with AKI had pre/post-HD NT-proBNP levels and were studied in a previous report by the authors (9). All patients were either already on chronic HD in case of established ESKD, were newly diagnosed with ESKD and initiated on HD upon admission or required HD due to AKI. All patients required volume removal during their inpatient HD. Of these 41 patients, 20 patients had pre/post-HD hs-cTnT and are the subject of this analysis, **Figure 1**. Two patients were diagnosed with ACS. None of the patients included in this analysis had AKI.

HD was done utilizing Fresenius 2008T dialysis machines (Bad Homburg, Germany: Fresenius Medical Care). Single-use high-flux Optiflux 160 or Optiflux 180 dialyzers were used (Fresenius Medical Care). Optiflux 160 dialyzer has a surface area of 1.5 m^2^, and ultrafiltration coefficient of 61. Optiflux 180 dialyzer has a surface area of 1.7 m^2^, and ultrafiltration coefficient of 76. The data were not stratified based on the dialyzer type as the difference in surface area is only 0.2 m^2^ between the two dialyzers used. Hs-cTnT was analyzed using electrochemiluminescence immunoassay (ECLIA) Elecsys Troponin T hs (Basel, Switzerland: Roche Diagnostics). The measuring range for hs-cTnT is 3 to 10,000 ng/L (pg/ml). The values below the limit of detection are reported as <3 ng/L, while the values above the upper measuring range are reported as >10,000 ng/L, or higher if the samples are diluted with Diluent MultiAssay. The coefficient of variation is less than 10% at the 99^th^ percentile of the reference range. The reference range is 0–14 ng/L for females, and 0–22 ng/L for males. The 99^th^ percentile upper reference limit is 14 ng/L for females, and 22 ng/L for males. These values are the diagnostic threshold for the diagnosis of ACS. Values are expressed as mean ± standard deviation (SD) when appropriate. Certain values are reported as median with range in parentheses. Differences between groups were analyzed with Wilcoxon signed-rank test, a non-parametric statistical test.

## Results

**Table 1** summarizes the baseline characteristics and the demographics of the study population. The main findings of the study are summarized in **Table 2**. Residual renal function in study patients was not assessed. Mean age was 60 years with range (32-86) years. The duration of HD in hours varied from 3.0-4.0 (mean 3.4). Volume removal in liters varied from 1.0–5.0 (mean 2.4).

**Table 1:** Baseline demographics and clinical characteristics of the study subjects.

Baseline Characteristics – ESKD Cohort (N = 20)	
Characteristic	Value
Age (years), mean ± SD	60 ± 15.6
(range)	(32–86)
Sex	
Male, n (%)	12 (60%)
Female, n (%)	8 (40%)
Race	
White (W), n (%)	7 (35%)
Black (B), n (%)	9 (45%)
Hispanic (H), n (%)	4 (20%)
Time on Hemodialysis (months)	16 (0–60)
Mean (range)	
Comorbidities	
History of CHF, n (%)	4 (20%)
Hypertension, n (%)	12 (60%)
Diabetes Mellitus, n (%)	4 (20%)
Pulmonary Edema on presentation, n (%)	6 (30%)
Coronary Artery Disease (CAD), n (%)	2 (10%)

W, White; B, African American; H, Hispanic; HD, hemodialysis; CHF, congestive heart failure.

For “time on HD,” a value of 0 indicates that ESKD was diagnosed and HD initiated at the time of hospital admission. This was the case in 3 of 20 patients.

**Table 2:** The main findings of the study. IQR is interquartile range.

Finding	ESKD N=20
Duration of HD session in hours Mean ± SD	3.5 ± 0.7
Volume removal in liters Mean ± SD	2.4 ± 0.9
Urea Reduction Ratio Mean ± SD	64.7±7%
Number of hospitalizations after the initial presentation during the study period. Median (range)	2.0 (0-6)
Mortality over the study period Number (%)	8 (40%)
Time from initial presentation until death in months. Median (range)	10.5 (1-21)
Pre-HD NT-ProBNP (pg/ml) Mean ± SD (range)	30,443 ±9,576 (1,543-35,000)
Post-HD NT-ProBNP (pg/ml) Mean ± SD (range)	29,569 ±10, 570 (1,316-35,000)
Pre-HD hs-cTnT (range) Median (IQR)	(14-1,236) 126 (154)
Post-HD hs-cTnT (range) Median (IQR)	(12-1,526) 155 (234)

NT-proBNP Age-Adjusted Diagnostic Cutoffs for Heart Failure: Age < 50 years: NT-proBNP ≥ 450 pg/mL, Age 50–75 years: NT-proBNP ≥ 900 pg/mL, Age > 75 years: NT-proBNP ≥ 1,800 pg/mL. High-Sensitivity Troponin-T (hs-cTnT) Reference Limits: Females: 0–14 ng/L (upper reference limit: 14 ng/L), Males: 0–22 ng/L (upper reference limit: 22 ng/L). The upper reference limit for hs-cTnT represents the 99th-percentile cutoff for normal values.

**Figure 2:**
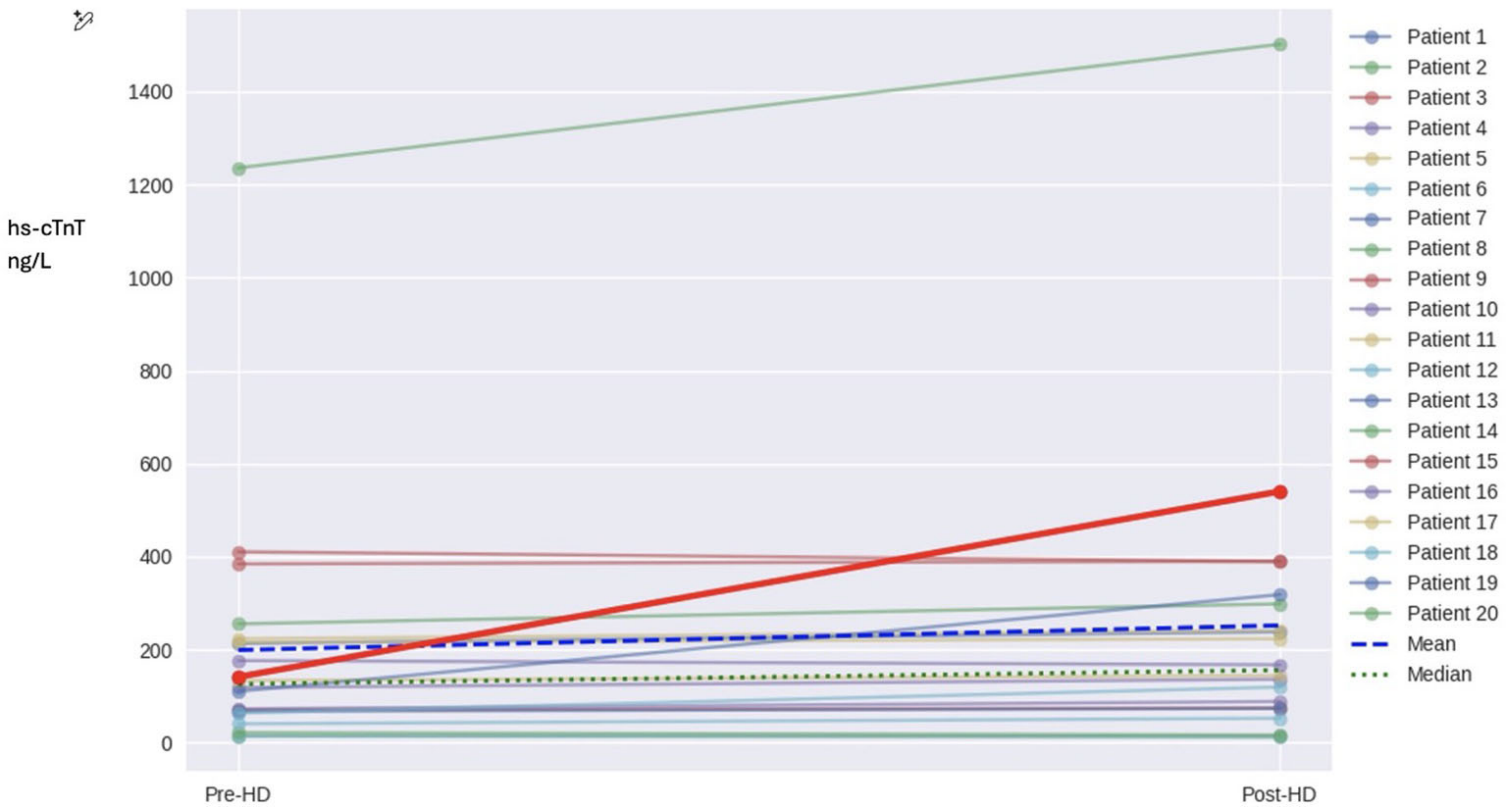
Spaghetti plot of pre-HD and post-HD hs-cTnT measured in ng/L for individual patients. The mean and median are plotted.

A hs-cTnT level was obtained on admission in every patient of the cohort to rule out ACS due to hypervolemia and dyspnea. Only 2 of 20 patients were diagnosed with ACS by the cardiology service. Neither patient had chest pain on presentation. The first patient was a woman who presented with dyspnea and had non-ST elevation myocardial infarction (NSTEMI). Hs-cTnT levels were significantly elevated, pre-HD level was 1236 ng/L, and post-HD it rose to 1526 ng/L. These values were outliers in the pre-HD and post-HD datasets, **Figure 2**. The second patient was also a woman who presented with pulmonary edema and had ST elevation myocardial infarction (STEMI), hs-cTnT pre-HD was 119 ng/L and rose to 310 ng/L post-HD. Only 3 of 20 patients (2 women, 1 man) had hs-cTnT levels within the normal range both before and after HD. The other 17 patients (85% of the cohort) had elevated levels pre-HD and post-HD. Mortality was 40% in the cohort; 8 of 20 patients died during the 3-year study period. Cause of death in 5 of 8 patients was cardiovascular disease, 1 died of sepsis, and 2 of complications of alcoholic liver cirrhosis. None of the three patients who presented with normal hs-cTnT levels died during the same interval.

NT-proBNP plasma levels were significantly elevated pre-HD in the cohort, the mean ± SD 30,443 ± 9,576 pg/ml, which is close to the upper limit of the test 35,000 pg/ml. Urea reduction ratio was generally satisfactory. Pre-HD hs-cTnT, range:14-1,236, median 126, IQR 154. Post-HD hs-cTnT, range: 12-1,526, median 155, IQR 234.

Application of the Shapiro-Wilk test to the pre-HD and post-HD datasets show that data do not follow normal distribution. Wilcoxon signed-rank test, a non-parametric statistical test, was used for statistical analysis. For n=20, and a two-tailed test at α = 0.05, p-value is 0.0042 which is statistically significant. Using the Hodges-Lehmann estimator to calculate the median differences between the two datasets, the 95% confidence interval (CI) is [-43.0, -4.0], this interval does not contain zero and supports the conclusion that the datasets differ significantly. The median difference is 9.5, the 95% bootstrap CI for the median difference is [4,18]. In other words, a single HD session with a high-flux dialyzer in an ESKD patient with hypervolemia increases plasma levels of hs-cTnT by an average of 25.6%, 51 ± 22, median 23 ng/L. In 16 of 20 (80%) patients the hs-cTnT levels increased, while they decreased in 4 of 20 (20%) patients.

## Discussion

Cardiac troponins are commonly elevated in patients with CKD in the absence of ACS. This elevation correlates with poor prognosis. In our cohort 17 of 20 patients (85%) had elevated hs-cTnT levels on presentation. Ooi et al. studied cTnT in 174 HD patients and found that 29% had levels above 100 ng/L, and 10% had levels above 200 ng/L (10). In 10 of 12 patients studied the levels of cTnT increased post-HD by an average of 140 ng/L. The cause of the increase was not clear and did not correlate with adequacy or duration of HD. Dierkes et al. followed 102 HD patients for 2 years or until their first cardiovascular event or death (11). cTnT was a strong predictor of mortality. Using a cutoff of cTnT of 100 ng/L, Deegan et al. studied 73 chronic HD patients (12). Of this cohort, 20 of 73 patients had cTnT levels ≥100 ng/dl. At 15 months 65% of those patients died, while only 15% of the patients with cTnT levels <100 ng/dl died. Elevated cTnT levels were associated with increased mortality. Abbas et al. studied 222 patients with CKD who were not on dialysis and who did not have ACS (3). cTnT was elevated in 43% of the cohort, including 20% of patients with CKD-3, 39% of patients with CKD-4, and 59% of patients with CKD-5. Elevated cTnT was associated with decreased survival. Apple et al. studied 733 ESKD patients to determine the predictive value of cTnT and cTnI (4). More patients had an increased cTnT (82%) compared to cTnI (6%) at the 99^th^ percentile. Mortality at 2-year was increased by 2- to 5-fold if both cTnT and cTnI were increased. The GUSTO IV trial (Global Use of Strategies to Open Occluded Coronary Arteries IV) showed that cTnT remained specific for ACS and that elevated levels correlate with poor prognosis regardless of creatinine clearance (7). A meta-analysis by Khan et al. involving 28 studies and 3931 patients concluded that elevated cTnT >100 ng/L is associated with increased mortality and high risk of cardiac death in ESKD patients even in absence of clinical symptoms (13). Mortality in our cohort was 40%, 8 of 20 patients died during the 3-year study interval. The US Food and Drug Administration (FDA) approved cTnT in ESKD patient for prognostication as a biomarker for mortality risk stratification.

The mechanism underlying elevated cTnT levels in ESKD patients remains unclear. It is unlikely to be due to poor clearance (6). Fredericks et al. analyzed plasma cTnT in 32 patients with ESKD before and post kidney transplantation and found that improved kidney function post transplantation had no impact on cTnT levels (14). cTnT was analyzed at 1,3,6, and 12 months post kidney transplantation. Ünlü et al. studied 70 HD patients and found that the increase in hs-TnT levels post-HD correlated with the rate of ultrafiltration. Ultrafiltration led to a decrease in left ventricular global longitudinal strain (indicating a reduction in cardiac contractility in a longitudinal direction) (15). Whether this increase in hs-cTnT is due to myocardial injury or stress resulting from rapid volume removal remains to be determined. In our data we found no correlation between ultrafiltration rate, or dialysis vintage and the level of rise in hs-cTnT, however, our sample size is small.

There is evidence that elevated cTnT in asymptomatic ESKD patient is indicative of subclinical myocardial injury (16). In a prospective study of 224 patients with ESKD on HD, DeFilippi et al. found that elevated cTnT levels were associated with increased mortality, cardiovascular events, and coronary atherosclerosis (17). There is a link between cTnT and left ventricular hypertrophy (LVH) in both HD, and peritoneal dialysis (PD) patients. This may lead to subclinical ischemia, and increased permeability of cardiac myocytes (18–21). deFilippi et al. performed cardiac magnetic resonance (CMR) in 26 asymptomatic HD patients with elevated cTnT levels (22). Evidence of myocardial infarction was present in a few patients. They speculated that other mechanisms such as uremic myocardial fibrosis may be responsible for the elevation in cTnT.

Our analysis shows that a single HD session with a high-flux dialyzer in patients with hypervolemia increased plasma levels of hs-cTnT by an average of 25.6%. This increase occurred in 16 of 20 (80%) patients. The molecular weight of troponin T of approximately 35 kDa makes it less likely to be filtered even by a high-flux dialyzer membrane. Studies regarding the effect of HD on cardiac troponin have yielded conflicting data. A study by Tun et al. in 145 asymptomatic HD patients showed no effect of HD on cardiac troponin I (23). Lippi et al. studied 34 HD patients and showed that a single HD session leads to a decrease in cardiac troponin T by 37%, and cardiac troponin I by 27% only with high-flux dialyzers (polysulfone or AN69) (24). Levi et al. found that HD with low-flux dialyzers reduced hs-cTnT by at least 10% (25). Wayand et al. studied the effect of HD in 59 patients over a one-year interval on cardiac troponin T and I. The exact percentage change for each marker was not specified (26). They concluded that dialysis leads to an increase in cardiac troponin T and a decrease in cardiac troponin I, irrespective of dialysis membrane used. In a one-year prospective study Gremaud et al. measured hs-cTnT monthly in 44 asymptomatic chronic HD patients (27). Hs-cTnT was elevated in almost all of the patients, the mean ± SD was 84 ± 59 ng/L. Most patients (84%) were on high-flux dialyzers. A single HD session led to a 14.5% reduction in the plasma levels of hs-cTnT. Thirteen patients had acute myocardial infarctions leading to a rise in hs-cTnT of >45% from their individual baseline measurement. The authors recommend checking hs-cTnT every 3–6 months to establish a baseline level for each patient. This will facilitate the diagnosis of ACS.

Since HD has the potential to influence hs-cTnT levels, baseline values should be obtained pre-HD (6, 26). Any rise from baseline in the post-HD period should be interpreted with caution (6, 27, 28). Canney et al. found that hs-cTnT was elevated in 76% of a prospective cohort of asymptomatic 1956 patients with CKD (median GFR 27 ml/min/1.73 m^2^) (29). They proposed a higher cutoff to improve the prognostic value of hs-cTnT. The cutoffs were 22.7 ng/L, 26.8 ng/L, and 35.5 ng/L, for GFR 30–44 ml/min, 20–29 ml/min, and below 20 ml/min respectively. Some ESKD patients have normal hs-cTnT. Any rise in hs-cTnT should be interpreted in a manner similar to non-ESKD patients. In our cohort 3 of 20 patients (15%) had normal hs-cTnT. Guidelines from the National Academy of Clinical Biochemistry in patients with ESKD and suspected ACS require a rise in troponin >20% within 9 hours for the diagnosis of acute MI (28).

Our analysis was done in patients with hypervolemia and provided data on the levels of NT-proBNP before and after HD. NT-proBNP was significantly elevated in the cohort and near the upper limit of the test. It is unclear whether the presence of significant hypervolemia has affected the levels of hs-cTnT.

Different studies have yielded conflicting results on the effect of hemodialysis on hs-cTnT levels. Our results differ from the results shown by Lippi et al. (24), and Gremaud et al. (27) which have shown a reduction in hs-cTnT post HD. The studies in the literature are heterogeneous, as they differ with regard to number, demographics, and co-morbidities of the subjects, type of analysis (retrospective vs prospective), type of dialyzer used, volume and rate of ultrafiltration, and timing of sampling. The American Heart Association (AHA) guidelines recommend a second troponin level 3 to 6 hours after the first sample (1). The National Academy of Clinical Biochemistry require a rise in troponin >20% within 9 hours in patients with ESKD for the diagnosis of acute MI (28). In our study the sample was taken 5.5 ± 0.75 hours after HD which essentially falls within guidelines-recommended interval. Because the timing of sampling could not be controlled retrospectively, earlier post-HD sampling was not feasible. Importantly, given the half-life of hs-cTnT, earlier sampling would be unlikely to convert an observed increase into an apparent decrease, particularly as no patient experienced an acute coronary syndrome between the end of dialysis and the time of the second measurement. Thus, the observed post-HD rise in hs-cTnT is unlikely to be an artifact of sampling delay. However, it may be prudent to obtain a second sample immediately after hemodialysis, and a third one if needed 6 hours later. This will assure greater consistency in future studies. It is conceivable in theory that hs-cTnT levels can increase due to fluid removal via ultrafiltration resulting in hemoconcentration or increase due to subclinical cardiac ischemia. A decrease in hs-cTnT can be attributed to clearance by dialysis or binding to the dialyzer. Clearance seems less likely because the molecular weight of troponin T is approximately 35 kDa and high-flux membranes clear molecules up to 20 kDa. Measurement of troponin T in the dialysate may clarify whether any dialyzer-mediated removal occurs.

Our analysis has several limitations. It is retrospective and observational in nature; therefore, hypothesis generating and cannot establish cause and effect. There is no control group. The absence of a control group limits the ability to attribute the observed changes solely to the dialysis procedure. A sample size of 20 is too small, which limits the generalizability of this study. Long-term data beyond the 3-year study period are not available. We did not utilize direct volume measurement such as bioimpedance for verification of volume expansion. Relying solely on radiographic and physical examination findings can be subjective. Certain parameters were not included in the analysis such as blood and dialysate flow rates, and anticoagulation use. Hs-cTnT was not measured in dialysate and hs-cTnT levels were measured only twice in each patient (pre- and post-HD). Residual kidney function was not assessed. The study design cannot provide predictions regarding morbidity and mortality. Long-term prospective studies with measurement of cardiac troponins in dialysate, and clarification of the effect of ultrafiltration volume and residual kidney function are needed. Data on possible adsorption of cardiac troponins to dialyzer membrane are also needed.

## Conclusion

In this retrospective analysis, hs-cTnT plasma levels were elevated at baseline in most patients with ESKD and hypervolemia. A single session of HD using a high-flux dialyzer was associated with a significant increase in hs-cTnT levels, with a mean rise of approximately 25.6%. These findings underscore the importance of obtaining pre-dialysis troponin measurements when evaluating ACS in ESKD patients. Interpretation of post-dialysis values should be cautious, particularly in ESKD patients with chronic troponin elevation. Baseline cardiac troponin levels and serial measurements may improve diagnostic accuracy and clinical decision-making. Further prospective studies are needed to determine the clinical significance and mechanisms underlying these changes.

## Data Availability

The raw data supporting the conclusions of this article will be made available by the authors, without undue reservation.
